# Isolated Mediastinal and Hilar Lymphadenopathy as a Manifestation of IgG4‐Related Disease Diagnosed by EBUS‐Guided Transbronchial Mediastinal Cryobiopsy

**DOI:** 10.1002/rcr2.70396

**Published:** 2026-01-07

**Authors:** Wen Zhang, Zansheng Huang, Lei Zhou, Hanxiang Song, Mingzhou Zhang, Guansong Wang, Ye Fan, Zhi Xu

**Affiliations:** ^1^ Institute of Respiratory Diseases, Department of Pulmonary and Critical Care Medicine Xinqiao Hospital, Army Medical University Chongqing China; ^2^ The First People's Hospital of Kaili Kaili Guizhou China; ^3^ Department of Pathology Xinqiao Hospital, Army Medical University Chongqing China

**Keywords:** cryobiopsy, endoscopic ultrasound, IgG4‐related disease

## Abstract

Immunoglobulin G4‐related disease (IgG4‐RD) is a fibroinflammatory condition that can involve multiple organs, including mediastinal lesions. Transbronchial mediastinal cryobiopsy has recently emerged as a novel sampling technique that improves diagnostic yield for mediastinal lesions, particularly in rare tumours and benign disorders, compared to endobronchial ultrasound‐guided transbronchial needle aspiration (EBUS‐TBNA). Here we report a case of IgG4‐RD presenting with hilar and mediastinal lymphadenopathy successfully diagnosed by transbronchial mediastinal cryobiopsy using a ø1.7 mm cryoprobe. The diagnosis was established in accordance with the 2020 revised comprehensive diagnostic criteria for IgG4‐RD, after exclusion of infectious and lymphoproliferative disorders. Endoscopic mediastinal cryobiopsy may represent a valuable and minimally invasive strategy for diagnosing mediastinal diseases.

## Graphical abstract

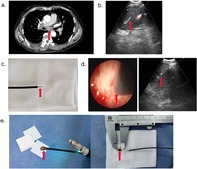

## 1 Introduction

Immunoglobulin G4‐related disease (IgG4‐RD) is an immune‐mediated fibroinflammatory disorder characterised by mass‐forming lesions, storiform fibrosis, and dense infiltration of IgG4‐positive plasma cells [1, 2]. Although serological and radiological findings are often non‐specific, clinicopathological correlation remains essential for diagnosis. According to the 2020 revised comprehensive diagnostic criteria, diagnosis of IgG4‐RD requires: (1) organ involvement, (2) elevated serum IgG4 levels and (3) characteristic histopathological findings, with exclusion of mimicking conditions such as infections or lymphoproliferative disorders [3].

Thoracic involvement is reported in up to 50% of IgG4‐RD patients [4, 5], manifesting as parenchymal lesions, pleural thickening, airway involvement, or mediastinal and hilar lymphadenopathy. Obtaining adequate mediastinal specimens is critical for diagnosis, yet standard Endobronchial ultrasound–guided transbronchial needle aspiration (EBUS‐TBNA) often yields insufficient cytologic material. Transbronchial mediastinal cryobiopsy, providing larger and better‐preserved tissue samples, offers improved diagnostic capability for both malignant and benign diseases [6].

Herein, we describe a case of mediastinal and hilar lymphadenopathy diagnosed as IgG4‐RD by transbronchial mediastinal cryobiopsy, fulfilling the 2020 diagnostic criteria, with systemic evaluation confirming no other organ involvement.

## 2 Case Report

**FIGURE 1:**
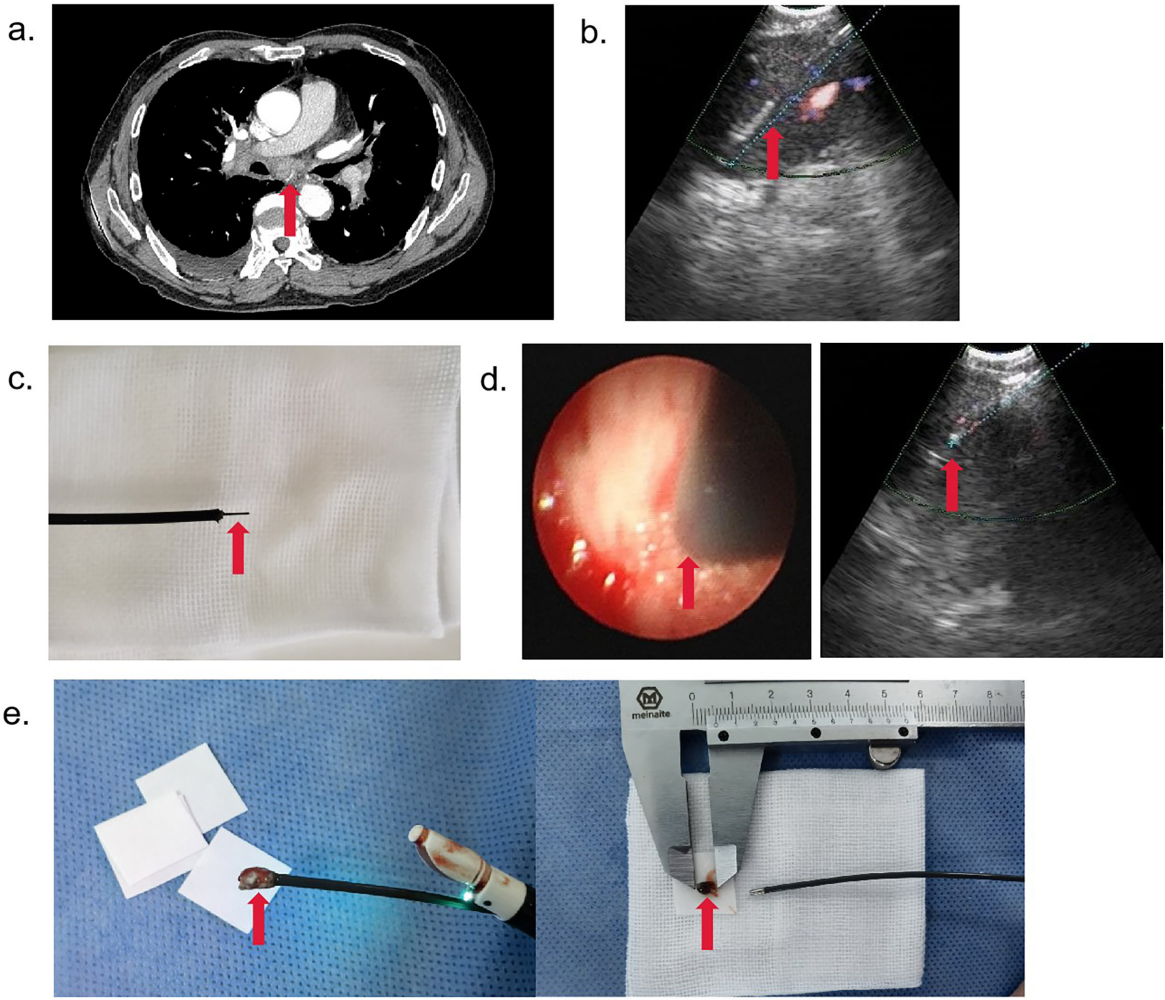
(a) CT image showing the paratracheal mediastinal lesion (Station 7, red arrow). (b) EBUS‐TBNA biopsy (red arrow) of the mediastinal lesion. (c) High‐frequency needle knife. (d) Cryoprobe (ø1.7 mm, red arrow) penetrating the lesion. (e) Sample (red arrow) obtained from transbronchial mediastinal cryobiopsy.

A 65‐year‐old male with an 80‐pack‐year smoking history presented with 1 month of cough and progressive dyspnoea. Contrast‐enhanced chest CT demonstrated bilateral hilar and mediastinal lymphadenopathy with right pleural effusion (Figure 1a). Biochemical analysis of right pleural fluid showed increased total protein and lactate dehydrogenase (LDH) levels, with a normal adenosine deaminase (ADA) level of 19.6 U/L, indicating exudate. Empirical anti‐tuberculosis therapy was initiated because of the high regional prevalence of tuberculosis and radiologic overlap with tuberculous lymphadenitis; however, treatment proved ineffective after 1 month. After the patient was enrolled in our hospital, laboratory tests showed elevated serum globulin 42.8 g/L (normal range 20–40 g/L), IgE 851 IU/mL (normal range 1–190 IU/mL) and IgG 31.9 g/L (normal range 7–15 g/L), with IgG4 5.34 g/L (normal range ≤ 2.0 g/L). Serum C3 0.46 g/L (normal range 0.9–2.1 g/L), C4 < 1.67 mg/dL (normal range 16–38 mg/dL), CA‐125 56.6 U/mL (normal range 0–35 U/mL), erythrocyte sedimentation rate (ESR) 68 mm/h (normal range 0–15 mm/h), C reactive protein (CRP) 45.9 mg/L (normal range 0–8 mg/L). Eosinophilia in bronchoalveolar lavage fluid was 4.4%. The peripheral blood eosinophilia and creatinine were normal. Autoimmune, including the titre of antinuclear antibodies, anti‐neutrophil cytoplasmic antibody (ANCA) and rheumatoid factor, and infectious markers were negative. Whole‐body CT and abdominal ultrasound showed no involvement of the pancreas, salivary glands, kidneys, or other organs. PET‐CT demonstrated increased uptake confined to the mediastinal and hilar lymph nodes. The patient was otherwise asymptomatic, and no hepatosplenomegaly was detected.

A diagnosis of sarcoidosis or mediastinal lymph node tuberculosis was initially suspected. No abnormality was found by regular bronchoscopy, and the patient did not receive preprocedure antibiotics. Pleural biopsy was not performed because the effusion volume decreased spontaneously and mediastinal sampling was prioritized. For further exploration, an EBUS bronchoscopy was performed. The patient received local anesthesia with 2% lidocaine and conscious sedation using midazolam 4 mg IV and sufentanil 6 μg IV. The paratracheal lesion (Station 7, measured approximately 3.1 × 2.1 cm) was localized according to contrast‐enhanced thoracic CT and EBUS bronchoscopy. Rapid on‐site cytologic evaluation showed nondiagnostic lymphoid material without malignancy. After four nondiagnostic TBNA passes with a 21‐gauge needle (Olympus NA‐201SX‐4021) (Figure 1b), a high‐frequency needle knife (Alton AF‐1810DZ, Figure 1c) was used to facilitate cryoprobe entry through the EBUS scope to the airway wall near the location of needle aspiration. Thereafter, the needle knife was applied and penetrated into the mediastinal lesion under real‐time ultrasound monitoring, which provided an entry to the mass (Figure 1b). The knife was withdrawn, and the flexible cryoprobe (OD: 1.7 mm, ERBE 20402–410) was then inserted into the target lesion under the visualized EBUS scope (Figure 1d). A 1.7‐mm cryoprobe was selected to obtain a larger core, as the lesion was firm and centrally located where a smaller probe might yield insufficient tissue. The cryoprobe tip cooled down to −60°C after 15 s with liquid carbon dioxide, and the attached rapidly frozen biopsy sample was gently extracted from the site. The cryoprobe was cooled for 15 s [7], longer than the standard 5–7 s suggested in recent evidence [8], to ensure adequate adherence in dense fibrotic tissue. The specimens (Figure 1e) were obtained by thawing in saline and fixed in formalin. Transbronchial mediastinal cryobiopsy was performed twice. The procedure took 15 min and was tolerable for this patient. No major complications were observed during the peri‐procedure and during 1‐month follow‐up.

**FIGURE 2:**
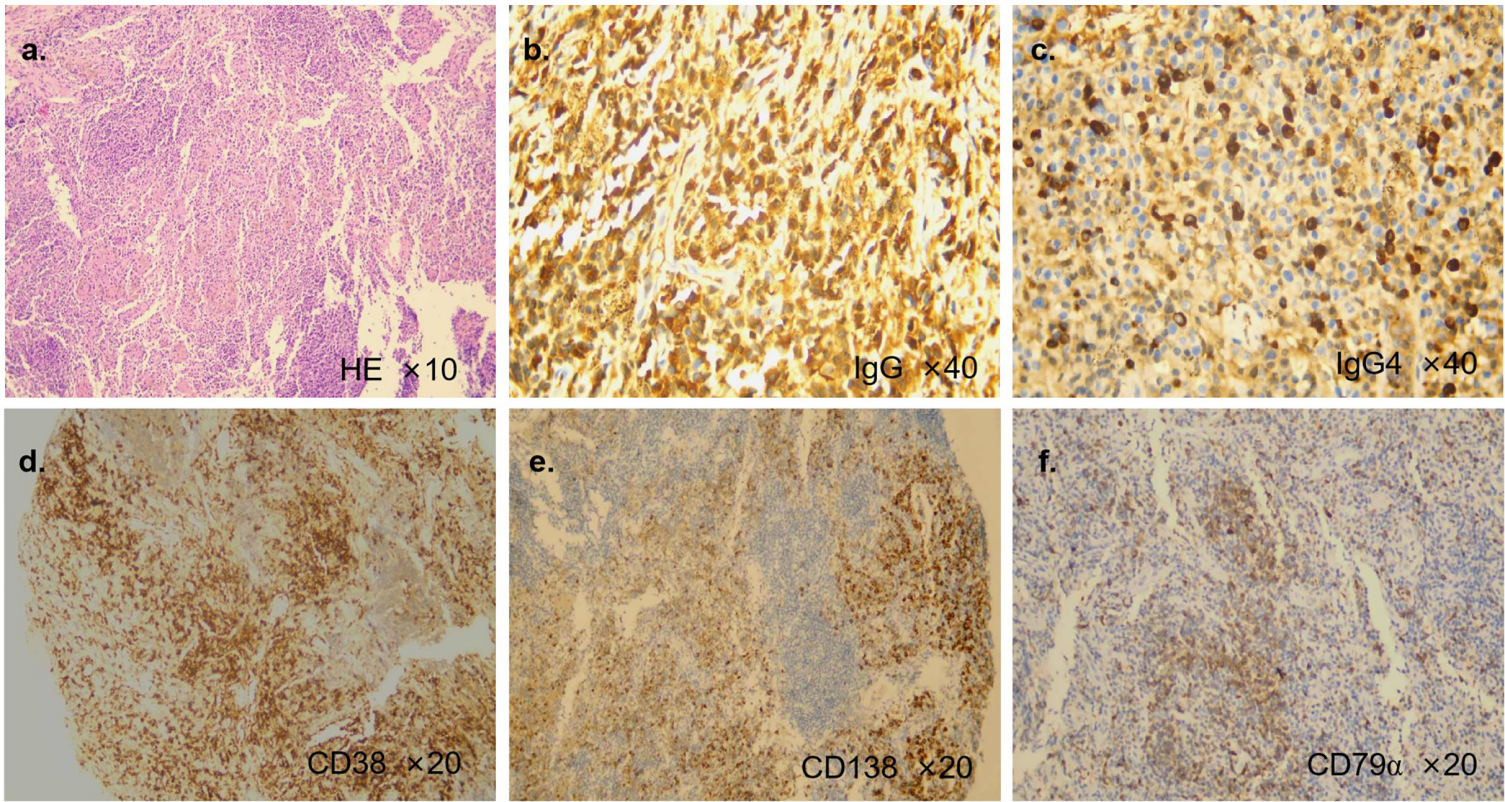
(a) H&E staining of the specimen from transbronchial mediastinal cryobiopsy. (b–f) Immunohistochemistry showing dense IgG4‐positive plasma cells with IgG4^+^/IgG ratio > 40%, consistent with IgG4‐RD.

The specimens from transbronchial mediastinal cryobiopsy showed lymphatic tissue without malignancy (Figure 2a). Immunohistochemistry staining indicated IgG^+^ (Figure 2b), IgG4^+^ (Figure 2c), CD38^+^ (Figure 2d), CD138^+^ (Figure 2e), CD79α^+^ (Figure 2f) and CyclinD1^−^. The patient was treated with oral prednisolone (1 mg/kg/day) and showed marked clinical and radiologic improvement over 6 months, supporting the diagnosis.

## 3 Discussion

IgG4‐related disease (IgG4‐RD) is a multisystem fibroinflammatory condition with diverse thoracic manifestations. When the disease presents as isolated mediastinal or hilar lymphadenopathy, distinguishing it from infections or lymphoproliferative disorders can be challenging because imaging and clinical findings are nonspecific [4, 5]. Therefore, histopathologic confirmation remains essential for diagnosis in accordance with the 2020 revised comprehensive diagnostic (RCD) criteria and the 2019 ACR/EULAR classification framework [1, 2, 3, 9, 10].

Endobronchial ultrasound–guided transbronchial mediastinal cryobiopsy (EBUS‐TMC) has recently emerged as an effective adjunct to conventional EBUS‐TBNA. Compared with TBNA, cryobiopsy retrieves larger and better‐preserved tissue cores that retain structural architecture crucial for diagnosing benign and rare diseases. Randomised and multicentre trials have shown that EBUS‐TMC significantly improves diagnostic yield, particularly for lymphomas, rare tumours, and non‐malignant conditions, while maintaining a favourable safety profile [3, 6]. The technique enables more reliable histopathologic and immunohistochemical evaluation, including accurate assessment of fibrosis and IgG4/IgG ratios, which are essential for differentiating IgG4‐RD from reactive lymphadenopathies [11, 12, 13, 14].

In our case, EBUS‐TMC provided sufficient diagnostic material after multiple nondiagnostic TBNA passes. The use of a 1.7‐mm cryoprobe and an extended 15‐s freeze was guided by the lesion's firm and fibrotic texture and its central location, ensuring adequate adherence and specimen size. Although recent studies recommend 5–7 s of freezing when using smaller (1.1 mm) probes [8], longer freeze durations may be beneficial for denser or fibrotic mediastinal lesions [7]. This case also suggests that the observed pleural effusion might represent a thoracic manifestation of IgG4‐RD. The diagnosis was supported by elevated serum IgG4 (> 2× ULN), compatible histopathology with > 40% IgG4^+^/IgG^+^ plasma cells, negative infectious workup, and a marked clinical response to corticosteroids, consistent with current diagnostic frameworks [1, 2, 3, 5, 13, 14, 15].

Glucocorticoids remain the first‐line therapy for IgG4‐RD and typically induce rapid symptom resolution and radiologic improvement [5, 12, 16]. Our patient achieved complete remission of mediastinal lymphadenopathy and pleural effusion after treatment with oral prednisolone (1 mg/kg/day) for 6 months. Relapse occurs in approximately 30%–40% of patients, emphasizing the need for long‐term follow‐up and, in some cases, maintenance therapy with immunosuppressants or biologics such as rituximab [14, 16]. In summary, this case demonstrates that EBUS‐guided transbronchial mediastinal cryobiopsy is a safe and minimally invasive diagnostic technique for thoracic IgG4‐RD, especially when conventional TBNA yields inadequate samples. Further multicentre studies are warranted to standardize procedural parameters—such as probe size and freeze duration—and to refine its role in the diagnostic algorithm for mediastinal diseases.

## Author Contributions

Wen Zhang, Zansheng Huang, Lei Zhou, Hanxiang Song, and Mingzhou Zhang contributed equally to this work. Wen Zhang, Zansheng Huang, Guansong Wang, Ye Fan and Zhi Xu were involved in the study design. Zansheng Huang, Yuhang Guo, Ping Wang and Ye Fan performed the procedure described. Wen Zhang, Zansheng Huang, Lei Zhou, Mingzhou Zhang and Hanxiang Song collected data. Hanxiang Song and Ye Fan analysed the data. Guansong Wang provided helpful guidance and suggestions. Wen Zhang wrote the first draft of the manuscript. Wen Zhang, Zansheng Huang, Lei Zhou, Hanxiang Song, Mingzhou Zhang, Guansong Wang, Ye Fan and Zhi Xu edited and approved the final version.

## Ethics Statement

This case was conducted ethically in accordance with the World Medical Association Declaration of Helsinki. The data collection was approved by the Ethics Committees of the Third Military Medical University (2019‐062‐01).

## Consent

The authors declare that written informed consent was obtained for the publication of this manuscript and accompanying images and attest that the form used to obtain consent from the patient complies with the Journal requirements.

## Conflicts of Interest

The authors declare no conflicts of interest.

## Data Availability

Data sharing not applicable to this article as no datasets were generated or analysed during the current study.
